# Validity of the Polar H10 for Continuous Measures of Heart Rate and Heart Rate Synchrony Analysis

**DOI:** 10.3390/s26030855

**Published:** 2026-01-28

**Authors:** Victor Chung, Louise Chopin, Julien Karadayi, Julie Grèzes

**Affiliations:** Laboratoire de Neurosciences Cognitives et Computationnelles (Inserm U960), Département d’Études Cognitives, École Normale Supérieure, Université PSL, 75005 Paris, France

**Keywords:** wearable sensor, hyperscanning, neurophysiological, autonomic, cardiac, interpersonal, dyad, emotion

## Abstract

**Highlights:**

**What are the main findings?**

- The Polar H10 chest strap showed almost perfect agreement with a reference Lead II ECG system for both individual heart rate measures (aggregate and moment-to-moment) and dyadic synchrony measures, with small systematic biases.
- Dyadic heart rate synchrony metrics derived from the Polar H10 were almost perfectly correlated to those from the Lead II ECG system, with no significant bias.

**What are the implications of the main findings?**

- The Polar H10 offers a valid, low-cost, and practical alternative to traditional ECG setups for studying both individual and interpersonal physiology.
- Although further testing is warranted, this could facilitate broader applications in cognitive, affective, and social neuroscience, enabling research to extend beyond tightly controlled laboratory settings into real-world environments.

**Abstract:**

Heart rate (HR), a non-invasive indicator of physiological arousal and autonomic nervous system engagement, is widely used in cognitive and affective sciences to monitor individual responses and interpersonal synchrony in dynamic emotional and social contexts. Recent advances in wearable sensors have enabled researchers to assess HR synchrony in ecologically valid settings. In this study, we replicate prior validations of the Polar H10 chest strap for individual HR measurement and extend these findings by evaluating its validity for measuring HR synchrony between individuals. Dyads completed a previously validated experimental task designed to elicit HR fluctuations while jointly attending to emotionally evocative, 5-min audiovisual stimuli. First, we observed high correspondence (Pearson’s *r* > 0.99) between the Polar H10 and a gold-standard ECG system in measuring individuals’ HR, both at the aggregate and moment-to-moment levels, thus confirming and extending prior findings. Second, we found high correspondence (Pearson’s *r* > 0.95) between the two systems in quantifying dyadic HR synchrony using multiple analytical approaches. These results support the use of the Polar H10 as a low-cost, easy-to-use, and reliable tool for both individual and dyadic HR measurement. This represents an important step toward establishing its applicability in real-world settings where traditional ECG systems are impractical.

## 1. Introduction

Heart rate (HR) is widely used in cognitive and affective sciences as it provides a non-invasive index of physiological arousal and autonomic engagement, offering a way to track how individuals respond to dynamic emotional and social contexts (e.g., [1,2]). Beyond individual responses, researchers have become increasingly interested in how HR patterns align across people, as such synchrony may reflect shared emotional, attentional, or behavioral states. For instance, a rapidly growing literature investigates the relationship between the synchrony of individuals’ HR and social outcomes such as the convergence of emotions (e.g., [3,4,5]) and group coordination (e.g., [6,7,8]).

**Figure 1:**
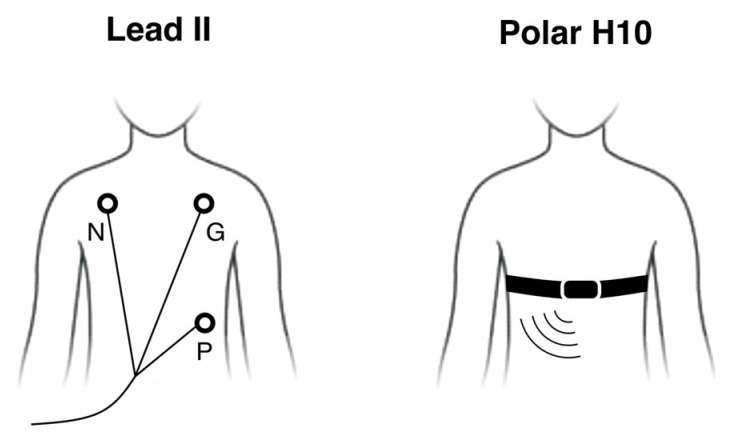
The two ECG systems compared in the study: the reference Lead II configuration combining wired surface electrodes (black circles; P: positive, N: negative, G: ground), and the wearable Polar H10 chest strap that connects wirelessly to an acquisition device.

Methodologically, the analysis of HR synchrony relies on the measurement of cardiac activity of several individuals simultaneously. In laboratory settings, this is typically accomplished using electrocardiography (ECG) with wired surface electrodes placed on the torso, such as in the Lead II configuration method (Figure 1). While these methods provide robust HR data, they are poorly suited for measuring HR in larger groups, both because of equipment costs and because the wired electrodes constrain participants’ movements. In contrast, recent advances in wearable physiological sensors have enabled researchers to quantify HR synchrony in dynamic, real-world contexts, including fire rituals, sporting events, concerts, and horror experiences, using devices such as the Polar belt [9], Zephyr BioHarness3 Model BH3 [10], Plux products [11], and Firstbeat Bodyguard 2 [12]. However, these studies applied wearable devices without explicitly testing their measurement accuracy. One exception is Milstein and Gordon [13], who validated the accuracy of the Empatica E4 wristband against mobile ECG in naturalistic dyadic interactions. The authors found strong agreement in inter-beat intervals (IBI, an inverse measure of HR), but they did not assess HR synchrony. Another study showed that the Wahoo Tickr chest strap and an established ECG system had similar accuracy in classifying patterns of HR synchrony [14]. However, the study did not directly compare the measurements of HR synchrony. Together, these observations highlight a methodological gap that the present study addresses by systematically evaluating the Polar H10 for continuous HR and HR synchrony measurement.

The Polar H10 [15] is a chest strap (Figure 1) that records ECG via three skin-contact sensors. The validity of the Polar H10 in measuring HR and HR variability has been established both at rest and during physical exercise by comparing it to gold-standard ECG systems [16,17] and photoplethysmography (PPG) systems [18,19]. These studies showed strong agreement between the Polar H10 and gold-standard ECG systems, with almost perfect to perfect correlations and very low systematic bias (smaller than 0.2 ms) between measurements of mean IBI. Furthermore, the Polar H10 demonstrated greater reliability and signal stability than PPG finger monitors in contexts involving participant movement [18]. Leveraging these advantages, more recent studies have employed Polar H10 devices to measure HR and quantify synchrony during group decision-making tasks [8,20].

Nevertheless, several questions remain regarding the suitability of the Polar H10 for measuring HR synchrony. While the device has demonstrated high validity in capturing HR and HR variability, its effectiveness for assessing HR synchrony hinges on its ability to accurately track the temporal dynamics of HR in real time, comparable to established reference systems. Previous research has compared the Polar H10 to gold-standard ECG measurement systems from an aggregative perspective, e.g., by averaging the participant’s HR over the experimental condition. However, strong agreement between measurements of HR at the global level of the recording epoch does not necessarily guarantee local alignment between continuous measurements of HR. Therefore, further research is needed to assess agreement between temporal, rather than aggregate, measurements of HR.

Furthermore, accurate HR synchrony measurement requires high reliability in accuracy across measurement devices. In lab settings, temporal discrepancies between devices are usually minimized thanks to wired connections and centralized time-keeping systems. By contrast, the Polar H10 uses Bluetooth Low Energy 5.0 for connection, and no commercially available system currently sets the time across multiple devices simultaneously. As a result, differences between the internal clocks of multiple Polar H10 units could introduce biases in synchrony measurements. Such biases may also depend on the method used to compute synchrony. For example, phase-lag measures of synchrony are particularly sensitive to the variability in the delay between HR measurements, whereas correlational indices may be less affected. Therefore, validation tests should compare the HR synchrony measurements obtained from reference systems with those derived from multiple Polar H10 devices used in combination.

Lastly, despite the relevance of the Polar H10 for measurements of HR and HR synchrony in a variety of socio-affective settings (e.g., [8]), its validation has so far been limited to comparisons during rest and physical exercise. However, changes in HR and HR synchrony often occur independently of exertion, such as in emotion-inducing contexts (e.g., [21]).

The present study extends prior evaluations of the Polar H10 in two novel ways: by assessing time-resolved HR accuracy across the recording time course and by assessing the accuracy of HR interpersonal synchrony. We used a previously developed experimental task that induces HR changes in dyads jointly attending to 5-min emotion-eliciting stimuli [22]. First, we hypothesized strong agreement between the reference and Polar H10 measurements of individuals’ HR. We expected to replicate previous findings on agreement in aggregate HR measures [17] and extend these results by demonstrating strong agreement in temporal HR measures. Second, we hypothesized strong agreement between the reference and Polar H10 measurements of dyadic HR synchrony computed using different analytical techniques.

## 2. Materials and Methods

### 2.1. Participants

Based on an a priori power analysis (*α* = 0.05 et 1 − *β* = 0.8), we determined a minimum sample size of 26 participants to detect heart rate changes from emotional videos (effect size *f* = 2.29) [22]. Participants were recruited via online posting with the following inclusion criteria: being 18- to 35-year-old, not suffering from auditory or visual impairment and reporting no history of neurological or psychiatric disorders, and speaking and reading French fluently. The final sample (*n* = 26 individuals, 18 F) had a mean age of 25.54 ± 4.61 years. Participants were randomly paired in 13 same-gender dyads of unacquainted individuals. The participants provided informed written consent and received monetary compensation (EUR 20) for their participation.

### 2.2. Experimental Procedure

To minimize motion- and speech-related confounds and to isolate device-related differences in cardiac signal acquisition and synchrony estimation, the experimental paradigm was intentionally designed to involve seated, non-verbal co-attendance under controlled laboratory conditions. Similarly to Ref. [22], participants assigned to the same dyad were welcomed separately. The participants read and signed an informed consent form and were equipped with ECG measurement devices (Polar H10, Lead II tripolar montage, Polar Electro, Kempele, Finland). The participants were seated side by side in a dimly lit, sound-attenuated room facing a common LCD monitor. The distance between the participants was approximately 60 cm, and the distance between the participant’s heads and the screen was about 1 m. After providing participants with the general instructions (e.g., avoiding intentional communication with the other participant), the two experimenters equipped the participants with headsets and left the room. During the experiment, participants completed scales and questionnaires one by one using individual keyboards. We used automatic audio cues indicating to participants when to answer and when to close their eyes, so that each participant’s answers remained confidential.

At the start of the experiment, participants were asked to report whether they knew the other participant and had seen them before (two binary-choice questions). Then, they were instructed to relax for five minutes while a fixation cross was displayed onscreen. Subsequently, three 5-min emotion-inducing videos were shown in a randomized order, each separated by a set of questions and a one-minute break. After each video, participants were asked whether they had previously seen the video (binary choice), and they self-reported their emotional experience during the video on the valence and arousal Likert scales (9-point) of the Self-Assessment Manikin (SAM) [23].

After viewing the third and final video, participants provided their sociodemographic information (age, gender, education level), followed by debriefing and compensation.

### 2.3. Stimuli

The three videos were chosen based on a previous study showing that they could induce distinct emotions [22]. Videos are widely used as emotional stimuli, notably when measuring interpersonal affective synchrony (e.g., [24]), as they evoke strong, long-lasting subjective and physiological responses [25]. The positive video was an excerpt from the French comedy film *Intouchables* [26]. The negative video was an excerpt from the documentary *Earthlings* [27], which depicts the mistreatment of animals in captivity. The neutral video was an excerpt from a video recording of a university library [28].

### 2.4. Cardiac Data Recording

The electrocardiogram (ECG) was continuously recorded during video presentation using both a reference measurement device and the wearable Polar H10 device. As a reference, we recorded ECG using three disposable AgCl electrodes in Lead II configuration. The signal was acquired at a sampling rate of 1000 Hz using a PowerLab 8/30 Data Acquisition System (ADInstruments, Sydney, Australia) and amplified prior to digitization (Dual BioAmp, ADInstruments). Simultaneously, the ECG was recorded with the Polar H10 chest strap [15] positioned on the lower part of the sternum beneath the pectoral region. The wearable device was connected wirelessly via Bluetooth 5.0 to an acquisition computer running a custom Python software (Python 3.0 [29]). This software was adapted from the “Bleakheart” software (v0.1.3) [30] and the Polar SDK API [31], enabling device connection, raw ECG signal recording, data visualization, and the insertion of event markers to delineate the experimental conditions. The software is openly accessible, along with a step-by-step tutorial [32]. Based on the device’s parameters, the ECG was sampled at 130 Hz ± 2%, and data were sent to the acquisition computer by packets of 73 samples and one timestamp corresponding to the last packet sample.

### 2.5. Cardiac Data Processing

#### 2.5.1. Individual Data

**Figure 2:**
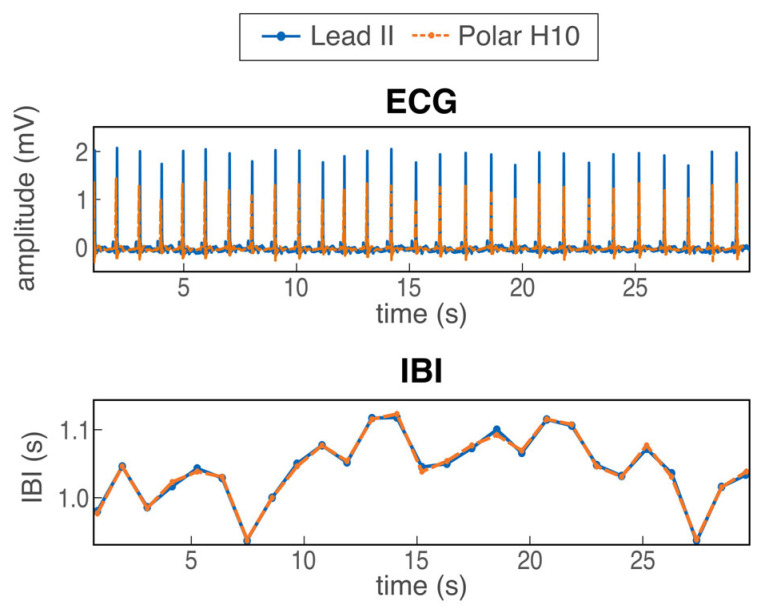
Example ECG data from one participant during the first 30 s of the negative video and the corresponding time series of IBI values.

Due to the variable sampling rate of the Polar H10, the ECG signal from the Polar H10 was spline-interpolated offline to obtain a continuous 130-Hz time series. We used the sensor’s timestamp as the time reference, and not the computer’s timestamp, so as to rule out errors due to the data transmission delay. Offline, the ECG signals from both measurement devices were then preprocessed similarly using custom software running on MATLAB (vR2019b) [33] with the FieldTrip toolbox (v20220104) [34]. First, we band-pass-filtered the signal between 1 and 60 Hz (Butterworth, first order) to remove drifting and alternative-current noise. Then, for each experimental condition, a template QRS complex was computed and convolved with the ECG. R-peaks were automatically detected as peaks in the normalized convoluted signal exceeding 0.6 standard deviations, and the ECG was visually inspected to correct for peak misidentification. The IBI was defined as the time distance in milliseconds (ms) between consecutive R-peaks, see Figure 2. Event markers were not perfectly aligned between the Lead II and Polar systems; hence, for each condition, we matched the first IBI values based on visual inspection (see Appendix A for further details). We then aligned the onset of the first R-peak in the Polar ECG to those in the Lead II ECG. For each participant and each experimental condition, we calculated the mean IBI.

#### 2.5.2. Dyadic Data

**Figure 3:**
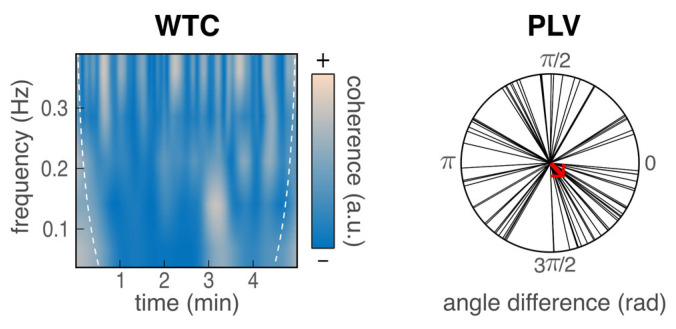
Example synchrony data from one dyad during the negative video, computed from transformed IBI time series; WTC: wavelet transform coherence; PLV: phase-locking value, which corresponds to the length of the mean unit vector (in red).

We estimated HR synchrony between participants as the phase-locking value (PLV) [35] and the wavelet transform coherence (WTC) [36] between individual time series of interpolated IBI values (cubic spline, 20 Hz). These methods are well suited to quantify time-resolved coupling between non-stationary signals such as HR time series, because they capture potentially meaningful phase- and frequency-dependent patterns. WTC was computed between band-pass-filtered signals in the known HR frequency range (0.04 to 0.4 Hz, Butterworth, fourth order). WTC values were averaged across time and frequency to obtain a time–frequency correlation value ranging from 0 (no synchrony) to 1 (perfect synchrony). PLV was computed on pairs of time series filtered in the high-frequency range (0.15 to 0.04 Hz) and Hilbert-transposed. PLV was defined as the absolute value of the mean difference in phase angle, ranging from 0 (no synchrony) to 1 (perfect synchrony) and representing the uniformity of time lag between participants’ IBI signals, see Figure 3. It is worth noting that the present study did not aim to study social interaction processes, in particular, to determine whether synchrony was driven by participants’ reciprocal interactions or by their exposure to common stimuli.

### 2.6. Statistical Tests

Analyses were conducted in MATLAB [33] and in R (v2025.05.1) [37] using RStudio (v4.5.1) [38]. For parametric statistical tests, we assessed the corresponding assumptions: normality (Shapiro–Wilk’s test), homoscedasticity (Levene’s test), relationship linearity (Ramsay’s RESET test) and the presence of outliers (Mahalanobis’ test). The results are available in the Supplementary Information S1. When these assumptions were not met, we used non-parametric tests instead, i.e., Spearman’s rank correlations and Wilcoxon tests. Effect sizes were converted into Pearson’s *r*. We set the alpha level at 0.05 for statistical inference, and we adjusted *p* values with Holm’s method to correct for family-wise error rate.

As a manipulation check, we used generalized linear mixed-effect models (GLMM, “lme4” R package, v1.1-37 [39]; “ordinal” R package, v2023.12-4.1 [40]) to examine the effect of the videos on self-reported valence (ordinal logistic regression), self-reported arousal (ordinal logistic regression), and mean IBI (linear regression). These variables were regressed on the fixed effects of the video (three-level categorical factor) and previous video exposure (binary categorical factor). The models included participants as random effects, unless it prevented model convergence.

To test our hypotheses, we evaluated the difference between the two measurement devices using Pearson’s product-moment correlation (r) and Spearman’s rank correlation (*ρ*), Lin’s concordance correlation (r_c_; “DescTools” R package, v0.99.60 [41]), and Bland–Altman bias, defined as the mean difference between measurements (“blandr” R package, v0.6.0 [42]). Compared to the product–moment correlation, the concordance correlation additionally captures the deviation of the best-fitting line from the identity line representing perfect concordance. The Bland–Altman mean difference provides a direct estimation of deviations or errors between the two measurements.

At individual level (Hypothesis 1), we compared mean IBI per condition by computing correlation, concordance, and bias for each condition across participants (*n* = 26). We also compared temporal measures of IBI by computing these indices between time series for each condition and participant, before calculating average correlation, concordance, and bias for each participant (*n* = 26). To assess whether the correlation varied as a function of time within each condition, we computed the Pearson’s product–moment correlation coefficient on sliding windows (size = 30 s, step = 15 s, *n* = 19) between the IBI time series for each participant and condition. For each condition, we then used a linear mixed-effect regression of Fisher’s z-transformed coefficients to model the fixed effects of the time window (continuous factor), the video (categorical factor), their interactions, and the random effects of participants. Post hoc, we computed the cross-correlation coefficient between the interpolated IBI time series to estimate the delay between measurement devices as the time lag maximizing the cross-correlation coefficient within a range of ±2 s (see Supplementary Information S2). At dyad level (Hypothesis 2), we compared synchrony indices by computing correlation, concordance and bias for each condition across dyads (*n* = 13), for PLV and WTC, respectively.

For both hypotheses, we assessed the effect size and significance of Pearson’s correlations at both individual and dyad levels by comparing the mean of the correlation coefficients (*n* = 26 individuals; *n* = 13 dyads) against zero using unilateral one-sided tests. We applied Fisher’s z transformation to the coefficients before any parametric test (Student’s *t*-test). To assess the effect size of the correlations, we use the following inference criteria: *r* = 0.3–0.5 for a “moderate” association, *r* = 0.5–0.7 for a “large” association, r = 0.7–0.9 for a “very large” association, and *r* > 0.9 for an “almost perfect” association. Lastly, we performed a unilateral two-sided *t*-test of Bland–Altman bias against zero.

## 3. Results

### 3.1. Manipulation Check

**Figure 4:**
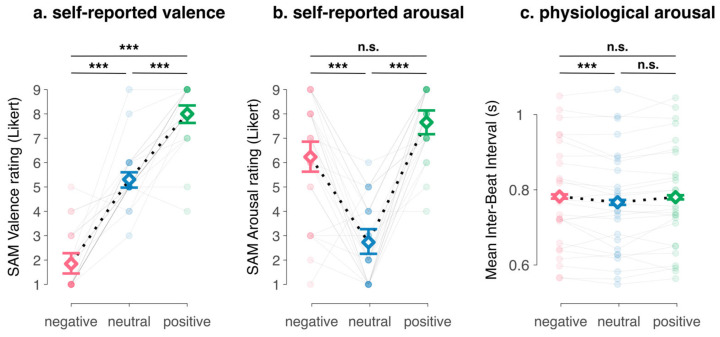
Effects of the experimental conditions on self-reported and physiological measures of emotion: (**a**) self-reported valence, (**b**) self-reported arousal and (**c**) physiological arousal indexed by the mean Inter-Beat Interval. Points represent participants’ scores; white diamonds represent sample means and error bars represent 95% confidence intervals around the mean, corrected for repeated measures. Stars indicate the significance of statistical tests of difference between means, corrected for multiple comparisons: *** *p* < 0.001; n.s.: non-significant.

As depicted in Figure 4, the regression models showed that the videos had significant effects on valence, arousal and mean IBI (see Supplementary Information S3 for regression tables). Participants were more likely to report more pleasant experiences during the positive (odds ratio = 33.26, 95%CI [6.01; 184.10], *p* < 0.001) and more unpleasant experiences during the negative video (odds ratio = 0.00, 95%CI [0.00; 0.04], *p* < 0.001) compared to the neutral video. We also found significant increases in self-reported arousal during the emotion-eliciting videos (positive vs. neutral: odds ratio = 91.29, 95%CI [14.76; 564.65], *p* < 0.001; negative vs. neutral: odds ratio = 27.24, 95%CI [7.28; 101.98], *p* < 0.001) and significant increases in mean IBI during the negative video (negative vs. neutral: *β* = 15.25, 95%CI [4.43; 26.06], *p* = 0.006) but not during the positive video (positive vs. neutral: *β* = 10.71, 95%CI [−6.08; 27.51], *p* = 0.208).

### 3.2. Individual-Level Aggregated Measurements

**Figure 5:**
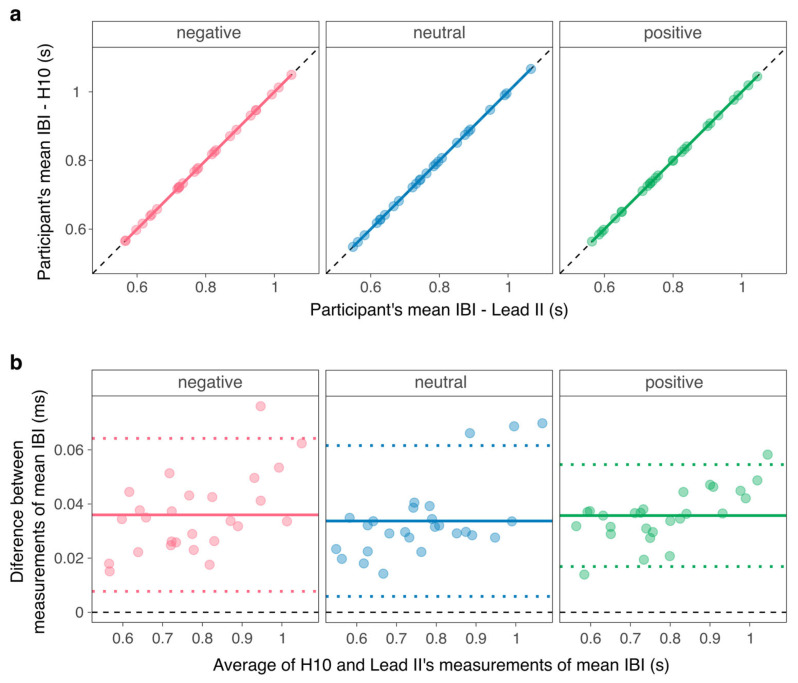
Agreement between measurements of participants’ mean IBI values per condition. (**a**) Correlation and concordance, where the black dashed line represents the line of perfect concordance and the colored line represents the regression line. (**b**) Bland–Altman plots, where the black dashed line indicates the null, the colored plain line represents the mean bias, and the colored dotted lines represent the limits of agreement. Points represent participants.

For each video, we found perfect correlations (all Spearman’s *ρ* = 1, *p_Holm_* < 0.001; all Pearson’s *r* = 1, *p_Holm_* < 0.001) and Lin’s concordance (all *r_c_*’s = 1, *p_Holm_* < 0.001) between the measurements of participants’ mean IBI (Figure 5a). The bias between measurements was small across videos (ranging from 0.01 ms to 0.08 ms), but one-sample Wilcoxon signed-rank tests showed that the bias was significantly different from zero for all conditions (Figure 5b): all effect sizes *r* = 0.87, *p_Holm_* < 0.001. Table 1 summarizes the data.

### 3.3. Individual-Level Temporal Measurements

**Figure 6:**
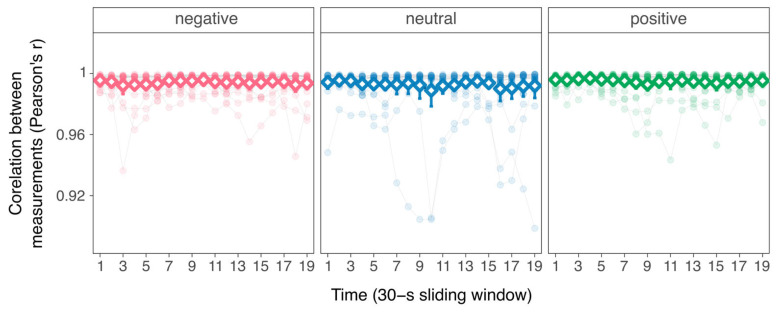
Correlation between the measurements of IBI time series as a function of time for each condition. Points represent participants’ Pearson correlation coefficients on sliding 30-s windows; white diamonds represent sample means and error bars represent 95% confidence intervals around the mean.

On average, the Polar H10 and Lead II IBI time series were perfectly correlated and concordant during the positive video (mean Pearson’s *r* = 1 ± 0; Lin’s *r_c_* = 1 ± 0), the negative video (mean Pearson’s *r* = 1 ± 0; Lin’s *r_c_* = 1 ± 0), and the neutral video (mean Pearson’s *r* = 1 ± 0.01; Lin’s *r_c_* = 1 ± 0.01). Although the bias between measurements was small across videos and participants (ranging from 13.93 to 76.12 nanoseconds), one-sample Wilcoxon signed-rank tests showed that the bias was significantly different from zero: all effect sizes *r* = 0.87, *p_Holm_* < 0.001. In addition, the regression of the time series’ correlation on sliding windows (Figure 6; see Supplementary Information S4 for regression tables) showed that Pearson’s correlation did not vary as a function of time during the positive video (*β* = −0.01, 95%CI [−0.02; 0.00], *p* = 0.103) or the negative video (*β* = −0.01, 95%CI [−0.02; 0.00], *p* = 0.230). In contrast, correlation slightly decreased as a function of time during the neutral video (*β* = 0.01, 95%CI [0.00; 0.03], *p* = 0.047).

### 3.4. Comparison of Dyad-Level Measurements

**Figure 7:**
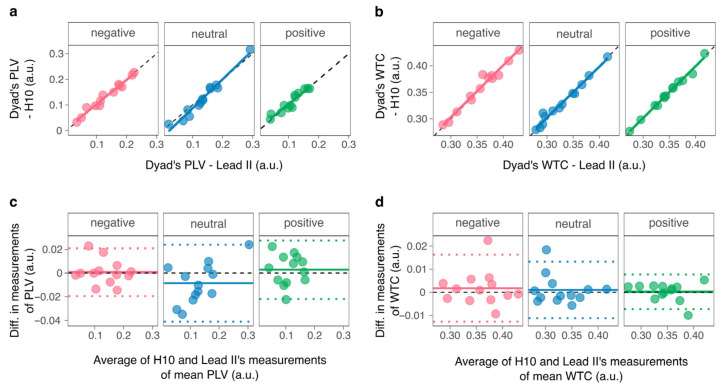
Agreement between measurements of synchrony per condition and index. Upper panels: correlation and concordance for (**a**) PLV and (**b**) WTC. Lower panels: Bland–Altman plots for (**c**) PLV and (**d**) WTC. In upper panels, the black line represents the line of perfect concordance, and the colored line represents the regression line. In lower panels, the black dashed line indicates the null, the colored plain line represents the mean bias, and the colored dotted lines represent the limits of agreement. Points represent dyads. a.u.: arbitrary unit.

For each video, and for both PLV (Figure 7a) and WTC (Figure 7b), the measurements of dyad’s synchrony were almost perfectly correlated (all Spearman’s *ρ* > 0.96, *p_Holm_* < 0.001; all Pearson’s *r* > 0.98, *p_Holm_* < 0.001) and concordant (all Lin’s *r_c_* > 0.95, 95% CI [0.85; 1]). The mean bias between measurements was not significant for PLV (Figure 7c; positive: effect size *r* = 0.22, *p_Holm_* = 0.91; negative: effect size *r* = 0.07, *p_Holm_* = 0.91; neutral: effect size *r* = 0.44, *p_Holm_* = 0.38) nor WTC (Figure 7d; positive: effect size *r* = 0.16, *p_Holm_* = 1; negative: effect size *r* = 0.20, *p_Holm_* = 1; neutral: effect size *r* = 0.11, *p_Holm_* = 1). Table 2 summarizes the data.

## 4. Discussion

Wearable devices that track heart rate (HR) provide a non-invasive window into autonomic nervous system activity and moment-to-moment physiological arousal, enabling researchers to study individual responses and interpersonal synchrony in dynamic emotional and social contexts. Among these devices, the Polar H10 chest strap stands out for its affordability, ease of use, and portability, making it particularly appealing for mobile and socio-affective research. However, despite its growing use, important questions remained: could it reliably capture fine-grained, temporal fluctuations in HR, and would the synchrony metrics from multiple devices match those obtained from gold-standard systems in social settings? The present study tackled these issues directly, showing that the Polar H10 provides valid measurements of both individual HR and dyadic HR synchrony, highlighting its potential as a practical tool for studying physiological dynamics in real-world contexts.

### 4.1. Measurements of HR

At individual-level, our findings replicate previous validation studies showing almost perfect concordance between HR indices derived from the Polar H10 and gold-standard ECG devices during both resting and exercise conditions [17]. Extending beyond aggregate indices, we examined moment-to-moment correlations between the two measurements of inter-beat intervals (IBIs) and likewise found almost perfect to perfect concordance.

Despite these high correlations, we observed a small but significant systematic bias—approximately 0.04 ms for aggregate measurements and in the range of nanoseconds for temporal measurements—which is smaller than biases previously reported during low-intensity exercise (0.1–0.2 ms, see [17]). Several technical factors likely contribute to this bias. First, slight variations in electrode placement between devices can alter QRS complex morphology, affecting peak detection. Second, the Polar H10′s lower temporal resolution, due to its 130 Hz sampling frequency, means that the R peak is often represented by a single data sample, limiting temporal precision.

Nevertheless, this bias did not lead to an increasing drift between the measurements over time. The average correlation remained stable across the session, except in the neutral condition. Here, we observed a significant decrease in the correlation between measurements as a function of time, though the correlation remained almost perfect. One plausible explanation is that the relatively faster heart rates observed in the neutral condition—significantly higher than during the negative video and showing a tendency to be higher than during the positive video—that were reflected in reduced IBI may have introduced greater variability or noise in the detection of R peaks. This explanation is consistent with prior findings suggesting that the accuracy of the Polar H10 decreases as HR increases [17].

### 4.2. Measurements of HR Synchrony

Crucially, the observed bias at the individual-level was small enough and did not accumulate to a degree that would compromise higher-level analyses, allowing us to turn to the central question of whether the Polar H10 can also provide valid measurements of dyadic HR synchrony in socio-emotional settings. Both phase-locking and time–frequency coherence metrics indicated almost perfect concordance between devices. Nonetheless, concordance between the two devices was slightly more variable for the phase-locking value (PLV) than for the wavelet transform coherence (WTC). This suggests that phase-locking measures may be more susceptible to small timing inaccuracies.

### 4.3. Limitations and Future Directions

There are several limitations to the present study. First, while the emotional videos reliably modulated subjective valence, arousal, and HR during the negative condition, the positive emotional condition did not elicit a significant change in HR. Consequently, the present study cannot determine whether the Polar H10 is sensitive to subtle HR changes associated with positive emotions, as our manipulation failed to elicit those changes. Second, all participants were seated and largely immobile; further research is needed to assess device validity under conditions involving movement and speech, which are characteristic of more naturalistic interactions (e.g., [16,17]). Third, our analyses focused on relatively short (5-min) time windows, although longer recordings may be more sensitive to time drift between devices, which could impact synchrony estimates. Finally, while we could circumvent the absence of a common time marker by aligning intra-participant ECG recordings, future applications should address the challenge of reliably aligning the time clocks of multiple Polar H10 sensors (see Supplementary Material S7 and [43]). In addition, our sample consisted of healthy young adults from Western, Educated, Industrialized, Rich, and Democratic (WEIRD) societies, which may limit the generalizability of our findings to other age groups, cultural contexts, or clinical populations.

## 5. Conclusions

Taken together, our results suggest that the Polar H10 provides reliable measurements of HR and HR synchrony. While minor biases exist, they are unlikely to compromise dyadic-level synchrony measures. Researchers should remain mindful of the device’s limitations, particularly with respect to sampling resolution, electrode placement, and conditions of elevated heart rate. Although further validation in dynamic social interactions is warranted, the Polar H10 could provide a low-cost, practical, and valid solution for investigating the neurophysiological correlates of group-level processes both in and beyond controlled laboratory settings.

## Figures and Tables

**Table 1:** Intra-participant comparison of IBI measurements per condition.

**Score**	**Negative**	**Neutral**	**Positive**
Lead II Mean IBI (ms)	782.07 (141.17)	766.82 (141.13)	780.29 (141.12)
Polar H10 Mean IBI (ms)	782.10 (141.18)	766.85 (141.14)	780.32 (141.13)
Spearman’s *ρ*	1	1	1
Pearson’s *r*	1 [1; 1]	1 [1; 1]	1 [1; 1]
Lin’s *r_c_*	1 [1; 1]	1 [1; 1]	1 [1; 1]
Bias (ms)	0.04 [0.03; 0.04]	0.03 [0.03; 0.04]	0.04 [0.03; 0.04]

Values in parenthesis indicate the standard deviation; values in brackets indicate the 95% confidence intervals around the mean.

**Table 2:** Intra-dyad comparison of synchrony measurements per condition.

**Index**	**Score**	**Negative**	**Neutral**	**Positive**
PLV	Lead II Score (a.u.)	0.13 (0.06)	0.13 (0.06)	0.11 (0.04)
	Polar H10 Score (a.u.)	0.13 (0.06)	0.13 (0.07)	0.11 (0.04)
	Spearman’s *ρ*	0.96	0.96	0.96
	Pearson’s *r*	0.99 [0.95; 1.00]	0.98 [0.94; 0.99]	0.95 [0.84; 0.99]
	Lin’s *r_c_*	0.98 [0.95; 0.99]	0.96 [0.91; 0.99]	0.95 [0.85; 0.98]
	Bias (a.u.)	0.00 [−0.01; 0.01]	−0.01 [−0.02; 0.00]	0.00 [0.00; 0.01]
WTC	Lead II Score (a.u.)	0.25 (0.04)	0.24 (0.04)	0.28 (0.05)
	Polar H10 Score (a.u.)	0.25 (0.04)	0.24 (0.04)	0.28 (0.05)
	Spearman’s *ρ*	0.99	0.99	0.99
	Pearson’s *r*	0.98 [0.95; 0.99]	0.99 [0.96; 1]	0.99 [0.98; 1]
	Lin’s *r_c_*	0.98 [0.95; 0.99]	0.99 [0.96; 1]	0.99 [0.99; 1]
	Bias (a.u.)	0.00 [0.00; 0.01]	0.00 [0.00; 0.00]	0.00 [0.00; 0.00]

Values in parentheses indicate the standard deviation; values in brackets indicate the 95% confidence intervals around the mean. a.u.: arbitrary units.

## Data Availability

The dataset and scripts supporting the findings of this study are available at https://doi.org/10.17605/OSF.IO/WYM3S (accessed on 22 January 2026).

## Supplementary Materials

The supporting information can be downloaded at https://doi.org/10.17605/OSF.IO/WYM3S (accessed on 22 January 2026), Table S1: Ordinal logistic regression model of self-reported valence; Table S2: Ordinal logistic regression model of self-reported arousal; Table S3: Linear regression model of mean inter-beat interval; Table S4: Linear regression model of sliding window correlation during the positive video; Table S5: Linear regression model of sliding window correlation during the negative video; Table S6: Linear regression model of sliding window correlation during the neutral video; Table S7: Descriptive statistics of normalized Euclidian distances; Figure S1: Example ECG from one participant during the first 30 s of the three experimental conditions (before correction of event marker timing); Figure S2: Example time series of interpolated IBI from one participant in the three experimental conditions.

## Abbreviations

The following abbreviations are used in this manuscript:

ECG	Electrocardiogram
HR	Heart rate
IBI	Inter-beat interval
PLV	Phase-locking value
r	Pearson’s correlation coefficient
r_C_	Lin’s concordance coefficient
ms	Milliseconds
s	Seconds
WTC	Wavelet transform coherence

## Appendix A

The Polar H10 records and transmits data to the acquisition device per packet of 73 samples, associated with a unique timestamp that corresponds to the last sample of the packet. Event markers are integrated into the Polar H10 data set by matching the marker to the first timestamp occurring after the event. As a result, event markers in the Polar H10 data set have a low temporal resolution, as they may truly align with any of the samples of the previous packet. In contrast, the system used to record Lead II ECG in this study involved a high-resolution event-marking system (StimTracker, Cedrus, San Pedro, CA, USA) that matches the marker to a data sample with millisecond accuracy.

Therefore, we took event markers from the Lead II measurement as our reference and only used the event markers from the Polar H10 measurement as a visual reference. Specifically, since both measurements were measuring the same physiological system (the participant’s heart), we assumed that the first IBI value after the Lead II marker should align with the first or second IBI values before the Polar H10 marker. Visual inspection enabled aligning the Polar H10 measurement to the Lead II measurement by minimizing the discrepancies between the first 10 IBI values of the experimental condition. In our study, the mismatch between the two measurements never exceeded two IBI values.
